# Fatigue and Impact Properties of Kenaf/Glass-Reinforced Hybrid Pultruded Composites for Structural Applications

**DOI:** 10.3390/ma17020302

**Published:** 2024-01-07

**Authors:** Thinesh Sharma Balakrishnan, Mohamed Thariq Hameed Sultan, Farah Syazwani Shahar, Adi Azriff Basri, Ain Umaira Md Shah, Tamer Ali Sebaey, Andrzej Łukaszewicz, Jerzy Józwik, Rafał Grzejda

**Affiliations:** 1 Department of Aerospace Engineering, Faculty of Engineering, University Putra Malaysia, UPM Serdang, Seri Kembangan 43400, Selangor, Malaysia; thinesh.sharma.ts@gmail.com (T.S.B.); farahsyazwani@upm.edu.my (F.S.S.); adiazriff@upm.edu.my (A.A.B.); ainumaira@upm.edu.my (A.U.M.S.); 2 Laboratory of Biocomposite Technology, Institute of Tropical Forest and Forest Product (INTROP), University Putra Malaysia, UPM Serdang, Seri Kembangan 43400, Selangor, Malaysia; 3 Aerospace Malaysia Innovation Centre (944751-A), Prime Minister’s Department, MIGHT Partnership Hub, Jalan Impact, Cyberjaya 63000, Selangor, Malaysia; 4 Engineering Management Department, College of Engineering, Prince Sultan University, Riyadh 11586, Saudi Arabia; tsebaey@psu.edu.sa; 5 Mechanical Design and Production Department, Faculty of Engineering, Zagazig University, Zagazig 44519, Sharkia, Egypt; 6 Institute of Mechanical Engineering, Faculty of Mechanical Engineering, Bialystok University of Technology, 15-351 Bialystok, Poland; 7 Department of Production Engineering, Faculty of Mechanical Engineering, Lublin University of Technology, Nadbystrzycka 36, 20-618 Lublin, Poland; j.jozwik@pollub.pl; 8 Faculty of Mechanical Engineering and Mechatronics, West Pomeranian University of Technology in Szczecin, 70-310 Szczecin, Poland; rafal.grzejda@zut.edu.pl

**Keywords:** plant fibre composites, structural composites, pultruded FRP profiles, mechanical properties

## Abstract

To address the weight, cost, and sustainability associated with fibreglass application in structural composites, plant fibres serve as an alternative to reduce and replace the usage of glass fibres. However, there remains a gap in the comprehensive research on plant fibre composites, particularly in their durability for viable structural applications. This research investigates the fatigue and impact properties of pultruded kenaf/glass-reinforced hybrid polyester composites tailored for structural applications. Utilising kenaf fibres in mat form, unidirectional E-glass fibre direct roving yarns, and unsaturated polyester resin as key constituents, pultruded kenaf/glass hybrid profiles were fabricated. The study reveals that pultruded WK/UG alternate specimens exhibit commendable fatigue properties (18,630 cycles at 60% ultimate tensile strength, UTS) and fracture energy (261.3 kJ/m^2^), showcasing promise for moderate load structural applications. Notably, the pultruded 3 WK/UG/3WK variant emerges as a viable contender for low-load structural tasks recorded satisfactory fatigue properties (10,730 cycles at 60% UTS) and fracture energy (167.09 kJ/m^2^). Fatigue failure modes indicate that the stress applied is evenly distributed. Ductile failures and delaminations during impact test can be attributed to damping and energy absorbing properties of kenaf fibres. Moreover, incorporating kenaf as a hybrid alternative demonstrates substantial reductions in cost (35.7–50%) and weight (9.6–19.1%). This research establishes a foundation for advancing sustainable and efficient structural materials and highlights the significant role of materials design in shaping the future of engineering applications.

## 1. Introduction

In an era of growing environmental concerns, the imperative for eco-friendly materials has never been more pressing. This pursuit becomes even more critical when considering the diverse industries that rely on materials with exceptional mechanical properties, durability, and sustainability. Exploring plant fibres as alternative reinforcements in composites offers a beacon of hope in pursuing materials that align with sustainability goals. This article delves into the intriguing fusion of two distinct yet complementary materials: kenaf (K) and glass (G)-reinforced pultruded composites targeted for structural applications. Pultruded composite profiles have been gaining increasing attention and are in significant demand in various industries. Pultrusion is a manufacturing process that produces continuous lengths of fibre-reinforced polymer (FRP) composite profiles with high strength and stiffness [1,2]. These profiles have excellent corrosion resistance, high durability, and low maintenance requirements, making them attractive for various applications [3,4]. Some industries with the potential for pultruded profile applications are demonstrated in Table 1.

Industries spanning automotive, aerospace, marine, transportation, and others all demand materials that strike a balance between strength, weight, and environmental impact. Traditional materials often fail to meet these requirements due to their energy-intensive production processes, limited renewability, and unfavourable disposal pathways. This prompted a shift towards alternative materials, and among them, plant fibres like kenaf, flax, hemp, jute, and advanced composites are gaining attention as they offer a unique synergy of properties. Kenaf, a rapidly growing plant with abundant availability and known for its fibrous stalks, has emerged as a promising natural reinforcement owing to its exceptional mechanical attributes, renewability, and biodegradability. In addition, combined with polymer matrices through pultrusion, kenaf fibres can yield lightweight yet strong composites with unique advantages for various industries [8]. Simultaneously integrating glass fibres into pultruded composites enhances stiffness, strength, and resistance to environmental factors, culminating in materials well-suited for demanding applications [9]. Hence, hybridising the strengths of kenaf and glass fibres in creating structural profiles through the pultrusion process holds the promise of harnessing the combined benefits of these materials.

Kenaf fibres present a compelling choice among plant fibres, such as jute, hemp, and flax, due to their distinct advantages. Notably, kenaf fibres offer a higher yield percentage, making them a more economical and sustainable option for various applications [10,11]. Their abundant availability in Southeast Asia further enhances their attractiveness as a readily accessible and regionally abundant resource. Additionally, kenaf fibres boast a strength-to-weight ratio that is comparable to other plant fibres, reinforcing their position as a versatile and cost-effective material for a wide range of industrial uses. While kenaf fibre composites share challenges common to other plant fibres such as high moisture absorption, low fire resistance, and modest thermal and mechanical properties, research has been conducted to overcome these limitations. Strategies include hybridization with glass fibres, incorporation of additives, optimum fibre orientations, ideal stacking sequences, and alkali treatments, all aimed at enhancing the overall performance and expanding the potential applications of kenaf fibre composites [12,13]. For instance, research by Muralidharan et al. [14] investigates the flame retardancy and thermal stability of kenaf fibre-reinforced epoxy composites, recognizing the inherent challenges in mechanical and thermal properties at elevated temperatures. Two composite laminates were fabricated, one with 6% NaOH alkali-treated kenaf mats and another with untreated woven kenaf mats, both containing 40% fibre weight fractions. The alkali-treated kenaf fibre composite exhibited V_0_ fire retardancy grade and demonstrated improved thermal stability. The treated kenaf composite demonstrated significant reduction in weight loss between 300 °C and 450 °C and a 13.6% increase in heat deflection temperature (HDT) compared to the untreated composite. Zakaria et al. [15] studied the impact of fibre orientation on the tensile response of kenaf fibre composites, comparing 0° and 45° orientations fabricated through pultrusion and filament winding methods respectively. The composite with 0° fibre orientation by pultrusion exhibits superior tensile strength (39.16 MPa) compared to the 45° orientation (18.97 MPa). Further improvement is achieved by creating a hybrid solid composite shaft, incorporating glass fibre reinforcement, resulting in a composite with tensile properties that are 96.29% superior to the kenaf shaft alone. The study underscores the significant enhancement in mechanical properties through the synergistic effect of hybridizing kenaf and glass fibres.

The durability of fibre-reinforced polymer (FRP) composites is assessed through environmental exposure testing, cyclic loading and fatigue tests, chemical resistance evaluations, thermal stability assessments, and microstructural analysis [16]. Among these tests, fatigue and impact testing are particularly crucial in durability analysis, as they simulate real-world conditions, assessing the composite’s resilience to repetitive loading and sudden impacts, which are common stressors in applications such as aerospace, automotive, and infrastructure, ensuring the material’s long-term reliability and safety [17]. Fatigue in FRP composites occurs through mechanisms such as matrix cracking, fibre breakage, interfacial damage, delamination, microcrack formation, and environmental effects [18]. These cumulative processes lead to progressive structural deterioration and a decline in mechanical properties over repeated cyclic loading. The Izod impact test assesses the impact resistance of composites, by measuring the energy absorbed during fracture when a notched specimen is struck by a swinging pendulum. In FRP composites, low-velocity impact mechanisms involve matrix cracking, fibre breakage, delamination, fibre pull-out, matrix ductility, crack arrest, and residual strength, all contributing to the material’s ability to absorb and dissipate energy during impact events [19].

Previous research has been conducted on kenaf fibre composites and kenaf/glass fibre hybrid composites, investigating their fatigue and impact properties. Asumani et al. [20] investigated kenaf fibre-reinforced polypropylene composites’ fatigue and impact strengths by manipulating the fibre loading in the composites and alkali concentration for fibre treatment. The findings indicated that 30 wt% of fibre loading and alkali concentration at 5–6% significantly improved kenaf composites’ fatigue and Izod impact properties. Feng et al. [21] studied the fatigue properties of fibre–metal laminate fabricated using kenaf, glass fibres, and aluminium metal. The results show that the fatigue sensitivity and strength are reduced when the kenaf-to-glass fibre ratio increases. They also stated that the K/G/K stacking sequence showed optimum fatigue resistance, hence suitable for low-load applications. Miah et al. [22] compared the durability properties of kenaf mat/unsaturated polyester and glass/unsaturated polyester composites after thermal ageing. The researchers recorded that although the kenaf-reinforced composites show lower mechanical strength than the glass-reinforced composites, they show stable low-cycle fatigue and better energy absorption properties when compared to glass fibre composites.

According to Supian et al. [23], hybrid kenaf/glass composite tubes with high kenaf fibre fractions have shown improved energy absorption values compared to pure glass fibre tubes. Owen et al. [24] stated that applying an epoxy coating to kenaf-reinforced acrylonitrile butadiene styrene (ABS) composites improved fatigue resistance. Their findings also demonstrated that the fatigue and impact strength of the epoxy-coated kenaf/abs composites increased at 200 °C of processing temperature when compared to 220 and 240 °C. Similarly, Al-Weily et al. [25] applied kenaf fibres to develop lower knee prosthesis. The kenaf-reinforced composite recorded a maximum fatigue life cycle of 18.35 × 10^5^ cycles, which satisfies the safety requirements for the biomedical device.

Researchers also investigated the hybridization effects on the durability of pultruded hybrid composites. According to Xian et al. [26], two types of carbon/glass fibre hybrid plates, namely fibre random hybrid (RH) and core–shell hybrid (CH) modes, were developed to enhance the mechanical properties of pultruded fibre-reinforced polymer composites. Through sustained bending loading and extended water immersion tests lasting 360 days, the research revealed that the random fibre hybrid mode demonstrated synergistic effects between carbon and glass fibres, effectively relieving incongruous bearing behaviour and stress concentration at the fibre/resin interface. The RH plate exhibited significantly higher tensile and flexural strength compared to the CH plate, with maximum percentages of 51.3% and 39.7% after 360 days and demonstrated superior corrosive resistance over five years in typical bridge service environments. Another study conducted by Guo et al. [27] introduces a 22 mm diameter carbon/glass fibre reinforced pultruded rod, for bridge cable application. Tension–tension fatigue performances were investigated across stress levels of 0.33, 0.44, and 0.60, with a fixed stress ratio of 0.4. The hybrid rod exhibited a shift in fatigue failure mode from shell-core debonding to uniformly splitting at the carbon and glass fibre/resin interface, resulting in improved fatigue life, stiffness retention, and residual mechanical properties. The study also presents a reliable fatigue life prediction model, recommending a fatigue limit of 0.45 at a stress ratio of 0.4 for the hybrid rod in bridge cable applications to ensure longevity beyond two million fatigue cycles.

Although the virtues of kenaf and glass-reinforced hybrid composites are apparent, a gap exists in exploring their capabilities in structural applications. Their durability for structural applications remains a question despite the research. While numerous studies have investigated their properties and applications, the combined potential of these materials in pultruded profiles has yet to be comprehensively explored. This research aims to bridge this gap by developing pultruded profiles crafted from kenaf and glass fibres and delving into hybrid composites’ fatigue and impact properties for structural applications. The main goal of this study is to incorporate kenaf fibres into pultruded profiles by replacing conventional fibreglass mats [28]. The stacking sequence is chosen based on the practical possibility of a pultrusion setup. Other stacking sequences, for example, direct roving as the outer layer, would result in dimensional instability of the profiles and difficulties during the fabrication process. Incorporating kenaf by replacing fibreglass mats is expected to reduce the cost and the weight of structural profiles intended for moderate to low load structural applications. Besides that, from the investigation, kenaf/glass hybrid profiles are expected to display promising fatigue and impact properties to prove its feasibility in practical structural application. Utilising kenaf mats in composite industries is also safer when compared to glass fibres as it reduces harmful breathable glass particles in the working environment. Furthermore, the utilization of kenaf mats also improves the aesthetic appeal, providing a natural and visually pleasing brown plant fibre appearance to the profiles, which may be desirable in certain applications. Hence, the successful development of kenaf and glass-reinforced pultruded composites could pave the way for lightweight, durable, and sustainable alternatives to conventional materials, potentially transforming how industries approach material selection for structural profile applications [29].

## 2. Materials and Methods

Utilising kenaf fibres in mat form, unidirectional E-glass fibre direct roving yarns, and unsaturated polyester resin as key constituents, structural profiles of various cross sections were meticulously fabricated using the pultrusion process. Table 2 shows the mechanical and physical properties of the raw materials used in this investigation.

### 2.1. Materials

The materials used in this research comprise reinforcing fibres, thermoset resin, and surfacing veils. For the reinforcing fibres, chosen kenaf fibres were hybridised with E-glass fibres. Kenaf fibres were purchased locally in Malaysia, whereas glass fibres were purchased from China. The matrix material used for the study is selected to be unsaturated polyester resin. By combining E-glass fibres’ robustness with Kenaf fibres’ eco-friendly characteristics, our research aimed to create a composite material with enhanced mechanical properties and sustainability.

#### 2.1.1. Kenaf

**Figure 1:**
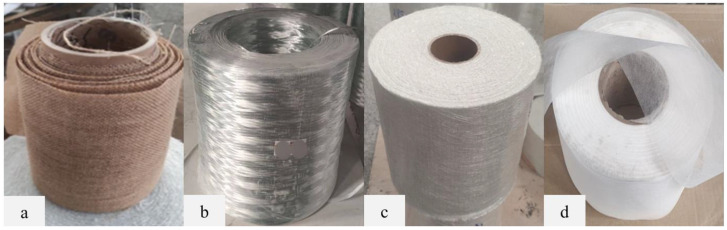
(**a**) Kenaf fibres (woven); (**b**) E-glass fibres (direct roving); (**c**) E-glass fibres (woven); (**d**) surfacing veil.

To fabricate plant/glass fibre-reinforced hybrid composites, kenaf fibres are purchased from Lembaga Kenaf dan Tembakau Negara (LKTN), Kota Bharu, Malaysia. Kenaf fibres are abundantly available in Malaysia, making them a readily accessible and sustainable reinforcement material for composite manufacturing. Figure 1a displays the woven kenaf fibre mat utilised in this study. The kenaf fibre mats were alkali treated at 5% sodium hydroxide (NaOH) at room temperature to enhance the fibre surface. Woven kenaf fibres ensure a well-organised and uniform distribution of the plant fibres within the composite, essential for enhancing mechanical properties and overall structural performance. Unlike kenaf fibres in yarn form, kenaf mats are found to be suitable for the investigation as the fibres did not break due to the exerted pulling force and no damages were done to the fibres due to the curing temperature during the fabrication. Besides that, one of the main goals of this research is to ideally replace woven glass fibre mats used in the pultruded composites industry. By incorporating locally sourced kenaf fibres, the composite fabrication process benefits from reduced transportation costs and supports the domestic agricultural industry, making it an eco-friendly and cost-effective approach to composite production.

#### 2.1.2. E-Glass

E-glass fibres in the form of both woven mats and direct roving were procured from Shandong Fiberglass Group Co., Ltd., Linyi, China. These fibreglass materials are commonly employed in the pultrusion industry to fabricate various cross-sections for pultruded profiles, primarily targeting structural applications. Figure 1b illustrates the E-glass fibres in direct roving form as continuous bundles of untwisted fibres. On the other hand, Figure 1c showcases E-glass fibres in stitched woven mat form, where the fibres are arranged in an organised and interlocked pattern. The availability of both direct roving and woven mat forms of E-glass fibres provides flexibility in the manufacturing process, allowing the production of various composite profiles with specific mechanical properties and performance characteristics. The use of high-quality E-glass fibres ensures consistency and reliability in the composite manufacturing process, contributing to the production of robust and durable structural components suitable for a wide range of industries.

#### 2.1.3. Unsaturated Polyester Resin

Unsaturated polyester resin is widely recognised for its economic viability in composite manufacturing applications. Unlike other thermoset resins, such as vinyl esters and epoxies, unsaturated polyester resin stands out due to its highly cost-effective nature. The affordability of this resin makes it a preferred choice for a wide range of industries seeking economical solutions for their composite needs. The type of UPR used in this study is Isophthalic, purchased from Eternal Materials Sdn Bhd, Pasir Gudang, Malaysia. Isophthalic UPR provides good mechanical, chemical-resistant, and water-resistant properties. The resin and hardener are mixed in the weight ratio of 10:1, as per the manufacturer’s specification. This precise mixing ratio ensures the resulting composite’s optimal curing and mechanical properties. By using unsaturated polyester resin in conjunction with plant and glass fibres, the research aims to develop environmentally sustainable and economically viable pultruded hybrid composites suitable for structural applications. This resin system’s cost-effectiveness and favourable mechanical properties make it a promising candidate for enhancing advanced composite materials’ commercial viability and widespread adoption.

#### 2.1.4. Surfacing Veil

Surfacing veils are well-regarded in composite applications for their unique benefits in enhancing composite materials’ surface finish and performance. Figure 1d shows the surfacing veils purchased from Utek Composite Co., Ltd., Changzhou, China, that were applied in pultruded composite fabrication. The surfacing veil is made of polyester material with a tensile strength of 132 N/50 mm and 53% elongation. The veil’s thickness is about 0.26 mm and weighs about 45 g/m^2^. Surfacing veils act as a protective layer, reducing surface defects such as porosity and fibre print-through that are common in pultruded composites. These veils also provide added corrosion resistance and improved UV resistance, preventing premature degradation and colour fading due to prolonged exposure to sunlight.

### 2.2. Pultruded Profile Fabrication

**Figure 2:**
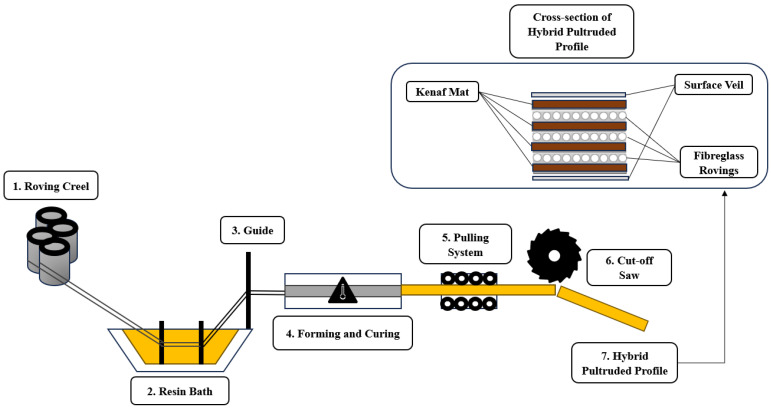
Pultrusion process and cross section of hybrid pultruded profile.

The pultruded profile fabrication was conducted through Mui Fatt Marketing Sdn Bhd, a local composites manufacturing company based in Port Klang, Malaysia. A hydraulic pultrusion machine was used to fabricate woven glass/unidirectional glass composites and woven kenaf/unidirectional glass hybrid pultruded profiles. Woven glass fibres and unidirectional glass rovings are conventionally used to manufacture fibreglass pultruded products. This research uses woven kenaf fibres to substitute conventional glass fibres and develop hybrid composites for pultrusion applications. Figure 2 shows the schematics of the pultrusion setup and cross-section of the fabricated hybrid pultruded profile.

**Figure 3:**
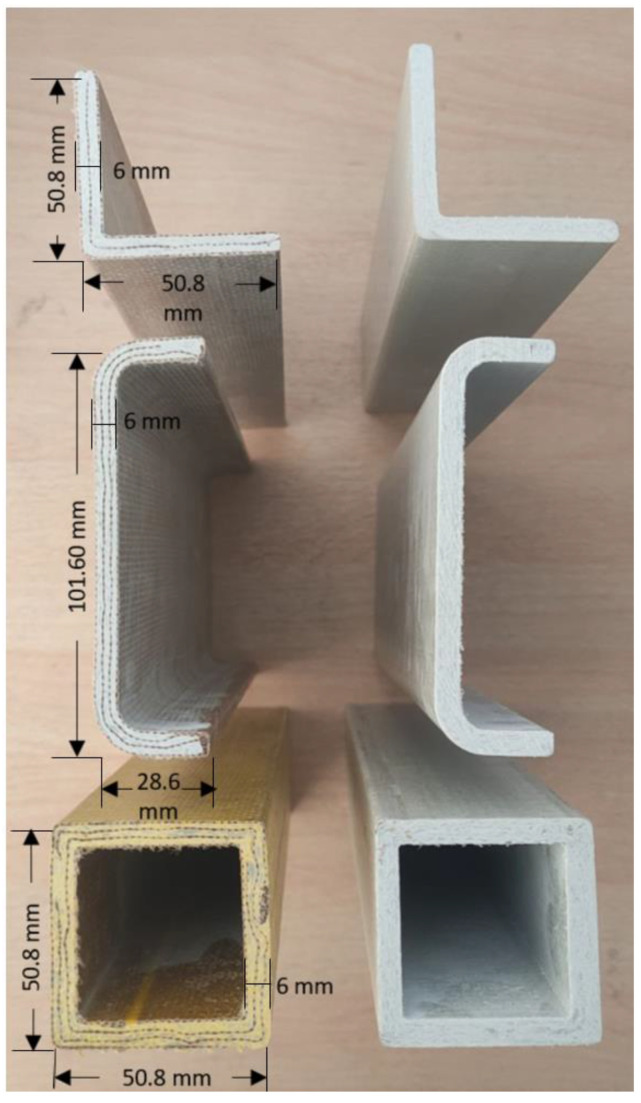
Pultruded WK/UG alternate profiles (**left**) and pultruded WG/UG alternate profiles (**right**) fabricated in 3 different cross-sections (angle bars, c-channels, and square tubes).

**Figure 4:**
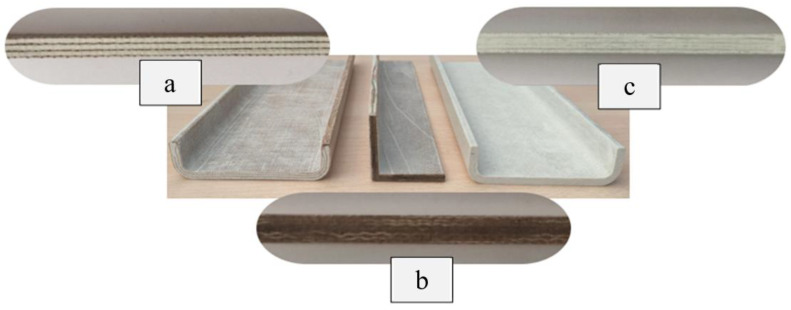
Stacking sequences of hybrid and non-hybrid pultruded profiles; (**a**) pultruded WK/UG alternate; (**b**) pultruded 3WK/UG/3WK; (**c**) pultruded WG/UG alternate.

The pultrusion process parameters such as pull speed (30 cm/min), pull force (6 Bar), and die temperature (159 °C) has been set optimally according to the kenaf fibre mats’ ability to withstand the force and temperature without being damaged [30,31]. The overall fibre/matrix ratio was fixed at 55/45 to achieve a high strength-to-weight ratio in the profiles [32]. The optimum fibre-to-matrix ratio typically ranges from 50 to 60% in pultrusion, as fibre content ratios less than 50% result in difficulties in curing and shaping the profiles. Fibre content exceeding 65% can lead to cracking in the fabricated profiles [33]. The profiles were controlled with seven layers of plies and a thickness of about 6 mm. The unsaturated polyester resin was used as the matrix, while dibenzoly peroxide (BPO) and tert butyl peroxy benzonate (TPBP) were used as hardeners to form the composite profile matrix. Pigments were added at 3% of the total resin weight to enhance the profile’s appearance and add colour. Calcium carbonate, CaCO_3_, and aluminium hydroxide, Al(OH)_3_, were used as fillers at 40% of the total resin weight [34]. Calcium carbonate serves as extenders to enhance mechanical properties and cost efficiency [35,36]. Conversely, aluminium hydroxide plays a dual role as both a flame retardant and extender [37]. The fillers are also added to improve surface finish and dimensional stability of the pultruded profiles [38,39]. The study design incorporates pultruded WG/UG as the control specimen, chosen for its widespread real-world applications. This specimen serves as the benchmark for comparison against the experimental kenaf/glass hybrid pultruded specimens. By contrasting the performance and characteristics of the kenaf/glass specimens with the established pultruded WG/UG, the study aims to discern any unique attributes or improvements offered by the novel composite. This comparative approach enhances our understanding of the potential advantages and practical viability of kenaf/glass pultruded materials in relevant applications. The fabricated profiles in 3 different cross sections for pultruded WK/UG alternate and pultruded WG/UG alternate are shown in Figure 3. The stacking sequences are demonstrated in Table 3 and Figure 4.

### 2.3. Experimental Testing

#### 2.3.1. Tensile Test

A tensile test was conducted for each group of kenaf/glass fibre hybrid composites and glass fibre composites. The tensile samples preparation and testing procedure was followed according to ASTM D-638 (Type I) with a crosshead speed of 5 mm/min. Tensile tests were conducted using the universal testing machine Zwick/Roell Z400 with 100 kN of load cell in the Faculty of Engineering, University Putra Malaysia, Seri Kembangan, Malaysia. The tensile specimen dimension was 165 mm × 19 mm × 6 mm. Five samples from each type of composite were tested, and the average value was determined and recorded. Tensile strength and tensile modulus were recorded. The ultimate tensile strength (UTS) values were used for fatigue analyses.

#### 2.3.2. Fatigue Test

The uniaxial fatigue test was conducted using a Fatigue Test Machine 8801, INSTRON, with 100 kN of load cell in CTRM Testing Laboratory Sdn. Bhd, Batu Berendam, Malaysia. Two specimens were prepared for each level of % ultimate tensile strength (UTS) for all types of composites. The testing procedure of the fatigue tests used in this study was based on ASTM D7791 (Type I), where the specimens were loaded under cyclic stress. The test was conducted at stress ratio, R = Q_min_/Q_max_ = 0.1, with a sinusoidal wave of a frequency at 20 Hz at a different applied stress level. Analysis was conducted at 23 ± 2 °C and relative humidity of 50 ± 5% room condition. The stress levels were set to 80%, 70%, 60%, and 50% of the UTS (Section 2.3.1). Table 4 shows the applied minimum and maximum stresses in fatigue testing.

#### 2.3.3. Izod Test

**Figure 5:**
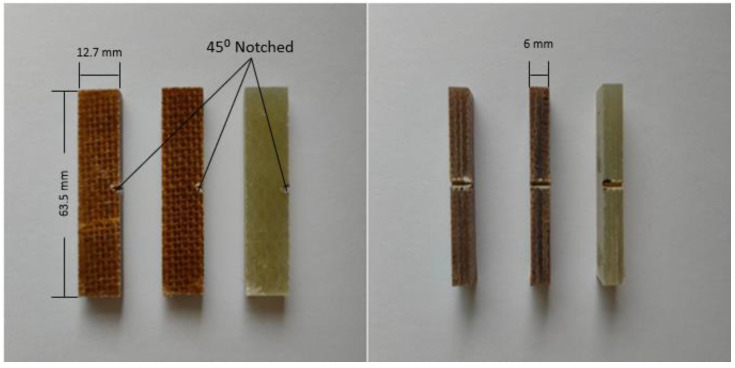
Izod samples.

The procedure of this test was conducted based on ASTM D256, Test C. The Izod impact test was conducted using an Impact Test Machine, CEAST 9050, INSTRON in CTRM Testing Laboratory Sdn. Bhd, Batu Berendam, Malaysia. The hammer energies range from 0.5 to 50 J. Five notched specimens of each type of composite were prepared, as shown in Figure 5. The specimens were then conditioned at 23 °C for 48 h after notching before testing. The test was then conducted at a laboratory temperature of 21 °C. The average value of the five repetitions was then recorded.

#### 2.3.4. Density Test

The density test was carried out according to ASTM D792. Three square specimens were prepared for each type of composite with a 20 × 20 × 6 mm dimension size. The specimens were conditioned at room temperature for two days before the density test. The testing used distilled water as its immersion liquid at room temperature (27 °C). The mass of the specimen in the air was first determined before immersing the specimen in the liquid. After immersion into the distilled water, its apparent mass was determined, and its specific gravity was calculated and recorded. The experiment was repeated three times, and the average value was recorded. The density data are used in the weight and cost analysis in Section 2.3.5. Density was calculated following Equation (1). Where 0.997 g/cm^3^ is the density of water at 25 °C.

Density of composite,

(1) $$\rho c=\frac{wc}{wc-ww}*0.997\;{g/\mathrm{cm}}^{3}$$

#### 2.3.5. Specific Strength, Cost, and Weight Analysis

The weight of the 0.3048 m (1 ft) square hollow pultruded tubes of the hybrid and non-hybrid composites is compared and analysed. The weight is obtained by multiplying the profile’s volume (3.277 × 10^−4^ m^3^) by the material’s density (Section 2.3.4). Weight of the pultruded hollow square tube was calculated as per Equation (2).

Weight = Volume of 0.3048 m (1 ft) profile ∗ Density (***ρc***) (2)

The raw material cost of reinforced fibres (kenaf and glass fibres) in a 0.3048 m (1 ft) square hollow pultruded tubes was calculated. The cost per kg of reinforcing fibres is multiplied with weight of reinforcing fibres used in 0.3048 m (1 ft) square hollow pultruded tube. The reinforcing fibre cost of the pultruded hollow square tube was calculated as per Equation (3).

Cost = Cost of fibres per kg ∗ Weight of fibres in 0.3048 m (1 ft) profile(3)

Specific strength, which is also known as strength-to-weight ratio of the materials, is calculated by dividing the strength of composites (Section 2.3.1) by its density (Section 2.3.4). Specific strength was calculated in the following Equation (4).

(4) $$\mathrm{Specific}\;\mathrm{Strength}=\frac{Ultimate\;Tensile\;Strength\;(UTS)}{Density\;(\rho c)}$$

## 3. Results and Discussion

As the main part of this experiment, the durability properties of hybrid and non-hybrid pultruded composites are analysed through fatigue and Izod impact testing. Each sample’s ultimate tensile strength (UTS) has been recorded from tensile testing, and the UTS data are used for fatigue analysis.

### 3.1. Static Properties

**Figure 6:**
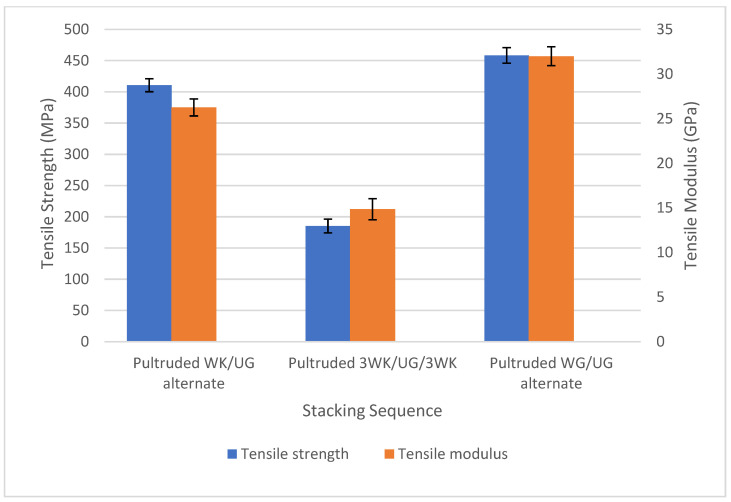
Tensile properties of pultruded composites.

Figure 6 shows the tensile strength and modulus of hybrid and non-hybrid pultruded composites. The standard deviation bars represent the standard deviations above and below the mean data. The non-hybrid, pultruded WG/UG alternate specimens have shown the highest tensile strength (458.28 MPa) and modulus (32 GPa) among the three pultruded laminates. This is due to the higher tensile properties of individual glass fibres compared to the kenaf single fibre. However, interesting results have been exhibited when the woven glass fibres are replaced with lower tensile strength kenaf fibres and hybridised with unidirectional fibreglass rovings. The hybrid pultruded WK/UG alternate specimens recorded a tensile strength of 410.6 MPa and a tensile modulus of 26 GPa. The samples recorded a strength reduction of 10.4% and a modulus reduction of 18.75% compared to pultruded WG/UG composites. However, hybrid specimen 3WK/UG/3WK has recorded the lowest tensile strength and tensile modulus at 185 MPa and 15 GPa, respectively. The higher kenaf to glass fibre ratio resulted in significant strength reduction at about 59.6% and modulus reduction at about 53.13% compared to the conventional fibreglass composites.

**Figure 7:**
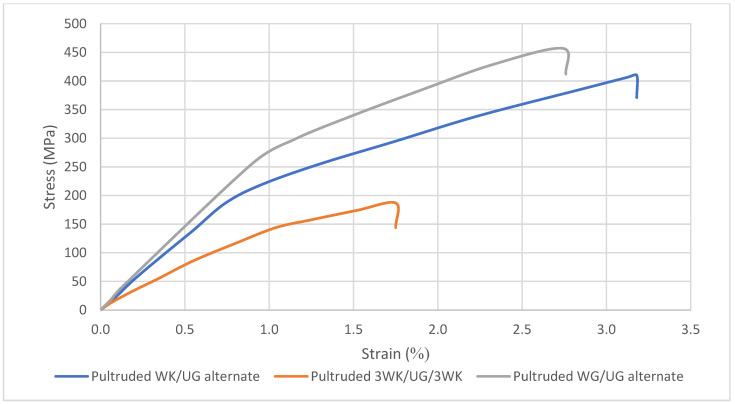
Tensile stress–strain curves of pultruded composites.

According to Figure 7, stress–strain profiles show the tensile properties of the hybrid and non-hybrid specimens. The graph shows a linear increment up to failure. The brittle behaviour of the composites is explained when the plot suddenly drops once the samples achieve ultimate tensile strength. When the peak load is achieved, all the samples fractured in a brittle behaviour. However, the maximum displacement of the specimens shows that pultruded WK/UG alternate is slightly more ductile in its plastic region when compared to pultruded WG/UG alternate specimens. The tensile modulus explains that pure fibreglass composite attained the highest stiffness compared to the hybrid specimens. However, among the hybrid samples, WK/UG alternate laminates showed better stiffness than 3WK/UG/3WK laminates. Overall, the load is applied in the direction of fibres, and the properties of single fibres determine the tensile strength and modulus of the composites. The experimental results indicate a decrease in tensile strength and tensile modulus as the volume percentage of glass fibres is decreased and replaced with kenaf fibres.

### 3.2. Fatigue Analysis

Repeated cyclic loading was applied to the composite specimens until fracture occurred. The stress intensity on the specimens was systematically decreased in 10% increments, starting from 80% of the ultimate tensile strength (UTS) and ending at 50% of the UTS. The force values corresponding to stress intensity and mean stress were calculated and imposed on the specimens. A plot of stress (S) versus the number of cycles to failure (N), known as an S-N curve or Wöhler curve, was generated to illustrate the test results. This curve represents the relationship between cyclic stress intensity and the number of cycles required for failure to occur.

**Figure 8:**
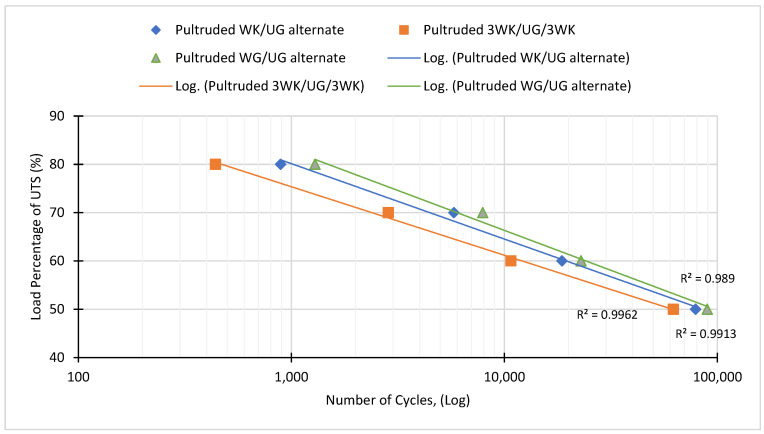
Fatigue test analysis of the pultruded composites.

By analysing the S-N curve in Figure 8, it becomes evident that decreasing the applied stress leads to an increase in the number of life cycles, indicating improved fatigue resistance. The hybridisation of kenaf and glass fibres and the varying ratio of kenaf to glass fibres in the hybridised pultruded composites significantly affected the fatigue life. This indicates notable differences in the fatigue properties between the hybridised pultruded composites and conventional pultruded composites made entirely of fibreglass.

Fatigue resistance is compared between the materials using Wöhler’s curve by analysing the stress intensity at a specific number of cycles to failure. The material with the higher stress intensity at a given number of cycles is considered to have a higher fatigue resistance. The data indicate that the pultruded WG/UG alternate achieves the highest fatigue resistance, followed by the pultruded WK/UG alternate and pultruded 3WK/UG/3WK specimen. Kenaf/glass fibre-reinforced hybrid pultruded composites achieved slightly lower fatigue resistance than non-hybrid specimens. Compared to glass fibres, kenaf fibres typically have lower tensile strength and fatigue resistance. Hence, increasing the proportion of kenaf fibres in the hybrid composite lowers the overall fatigue resistance of the material.

On the other hand, fatigue sensitivity between materials is analysed from the slope of the S-N curves. A steeper slope indicates a higher rate of fatigue resistance degradation, implying greater fatigue sensitivity. Materials with steeper slopes have a more rapid reduction in fatigue life as the stress intensity increases. The data show that the pultruded WG/UG alternate achieves the highest fatigue sensitivity, followed by the pultruded WK/UG alternate and pultruded 3WK/UG/3WK specimen. Notably, an increase in the kenaf-to-glass ratio has resulted in decreased fatigue sensitivity. Sharba et al. [40] stated that an increase in the kenaf-to-glass ratio in its hybrid composites resulted in a decrease in fatigue sensitivity, which is a desirable property of materials at low-load applications. A study on plant fibre composites by Liang et al. [41] showed similar properties where flax fibre reinforced composites show decreased fatigue sensitivity and strength compared to glass fibre composites.

Kenaf fibres, being of plant origin, possess inherent damping characteristics. Introducing kenaf fibres in the hybrid composite enhances damping properties [42,43]. Damping refers to the ability of a material to dissipate energy during cyclic loading, reducing the magnitude of stress fluctuations. This damping effect helps to absorb and dissipate energy, mitigating stress concentrations and reducing fatigue sensitivity. Therefore, kenaf fibres in hybrid composite exhibit lower sensitivity to fatigue-induced failure compared to fibreglass composites. Due to its lower elastic modulus and excellent damping properties, kenaf fibres exhibit improved damage tolerance compared to brittle glass fibres [44]. Hajer et al. [45] stated that plant fibre composites such as flax-PP, hemp-PP, and kenaf-PP demonstrated improved damping properties compared to glass fibre composites.

Similarly, findings from Diharjo et al. [46] show that an increase in the kenaf-to-glass fibre ratio led to an improved vibration-damping factor and a decrease in the elastic modulus of the hybrid composite. The increased kenaf content in the hybrid composite introduces additional mechanisms for energy absorption, enhancing the composite’s resistance to fatigue crack initiation and propagation [47]. The findings are supported by Sharba et al. [48] when kenaf fibre composites demonstrated a slower stiffness degradation when compared to glass fibre composites. Researchers have also recorded that woven kenaf fibres offer an excellent balance in static and fatigue resistances with low fatigue sensitivity in bidirectional planes compared to glass fibres, making them attractive in low load-bearing and moderate load-bearing structural applications [49].

Fatigue is typically categorised into two distinct regions: high-cycle fatigue and low-cycle fatigue. High-cycle fatigue manifests failure signs after exceeding 10,000 cycles under relatively low-stress levels. Conversely, low-cycle fatigue occurs at fewer cycles, usually below 10,000, accompanied by increased plastic deformation [50]. Upon examining the S-N curve, it is observed that when the stress level reaches 60% of the ultimate tensile strength (UTS), all the data points are situated within the high-cycle fatigue region. Hence, WK/UG alternate hybrid and 3WK/UG/3WK specimens demonstrate good fatigue resistance and durability, particularly in high-cycle fatigue conditions. The materials withstand a large number of cycles, exceeding 10,000, at relatively low-stress levels (below 50% of UTS, respectively) without signs of failure. This indicates that the material has the potential for long-term reliability in low to moderate load structural applications involving cyclic loading [51,52].

Table 5 depicts fatigue testing results, focusing on failure modes at 50%, 60%, 70%, and 80% of the fatigue life. Notably, all tested samples experienced failure due to a combination of fibre pull-out, fibre breakage, delamination, debonding, and matrix cracking. The severity of delaminations increased uniformly across all samples as the fatigue life decreased from 80% to 50%, indicating cumulative damage, progressive matrix cracking, intensified stress concentrations, and weakened interlaminar bonds as contributing factors to the escalating delamination severity. The observation suggests that the applied stress was efficiently distributed along the specimens, leading to significant delamination within the material. The kenaf/glass hybrid specimens exhibited unique failure modes that provide valuable insights into the interfacial adhesion between the kenaf fibres and the direct roving of glass fibres. Sufficient interfacial adhesion indicates good bonding and interaction between the less dense kenaf fibres and the direct roving. Proper wetting, which is the process of the resin impregnating the fibres during composite manufacturing, likely contributed to this enhanced interfacial adhesion [53]. The improved interfacial adhesion between the kenaf fibres and the direct roving is significant for composite performance.

Overall, different ratios resulted in varied fatigue performances, emphasising the importance of fibre composition in achieving desired durability. The hybridisation of kenaf and glass fibres introduced a new dynamic in the material’s fatigue behaviour. These hybrid composites demonstrated altered fatigue characteristics due to the combined properties of kenaf and glass fibres, resulting in different fatigue life cycles.

### 3.3. Impact Analysis

Izod impact testing is a mechanical test to evaluate a material’s impact strength or toughness. It measures the energy required to break a specimen under a single, high-velocity blow. This test is particularly relevant for assessing a material’s ability to withstand sudden impact or shock loading, a critical aspect of its durability in various applications. It involves striking a standardised notched specimen with a pendulum hammer and measuring the energy absorbed during the fracture. Structural applications often involve materials that must withstand impact or sudden loading conditions. The Izod impact test provides valuable information about a material’s ability to resist and absorb energy during impact events. These data are crucial for assessing the safety and reliability of structural components, as they help identify materials that can withstand unexpected or dynamic loading conditions without catastrophic failure.

**Figure 9:**
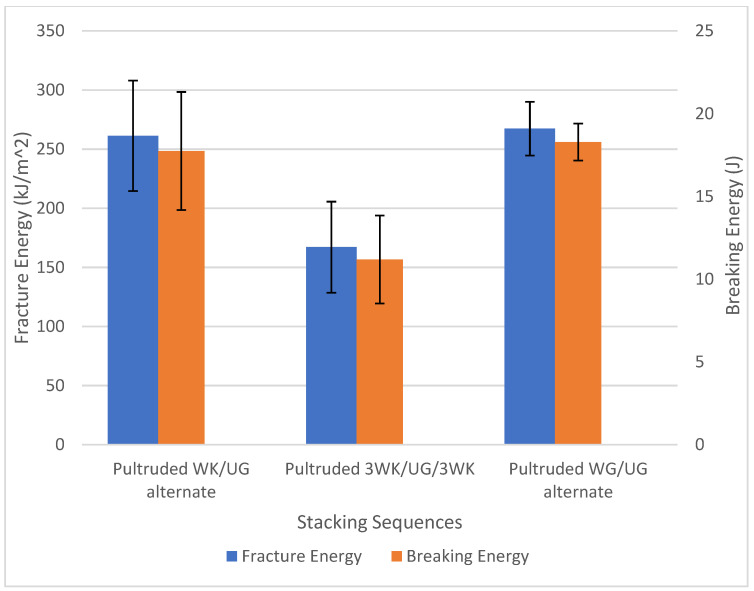
Fracture energy of pultruded composites.

According to Figure 9, the pultruded WK/UG alternate hybrid composites show similar fracture energy with pultruded WG/UG alternate composites at about 260 kJ/m^2^. Kenaf fibres and glass fibres possess different mechanical properties. While glass fibres are known for their high stiffness and strength, kenaf fibres offer good impact resistance and toughness [54]. Combining these two fibre types in hybrid composites can result in a complementary blend of properties. Singh et al. [55] recorded that hybridised kenaf/glass composites demonstrate significantly higher fracture energy than pure kenaf composites. Hence, the inherent impact resistance of kenaf fibres, combined with the strength of glass fibres, can contribute to a similar overall fracture energy compared to fibreglass composites. However, further increasing the kenaf-to-glass ratio resulted in a slight reduction in fracture energy. Similar discussions have been made on plant/glass fibre hybrid composites by Saroj et al. [56] and Ghani et al. [57], where the fracture energy reduced with a significant increase in the kenaf-to-glass ratio and jute-to-glass ratio, respectively. The fracture energy of the pultruded 3WK/UG/3WK is 37.5% lower than the pultruded WG/UG alternate. Hence, pultruded WK/UG alternate is found to have the optimum kenaf-to-glass ratio and stacking sequence as the composites demonstrate almost similar impact properties compared to conventional fibreglass.

**Figure 10:**
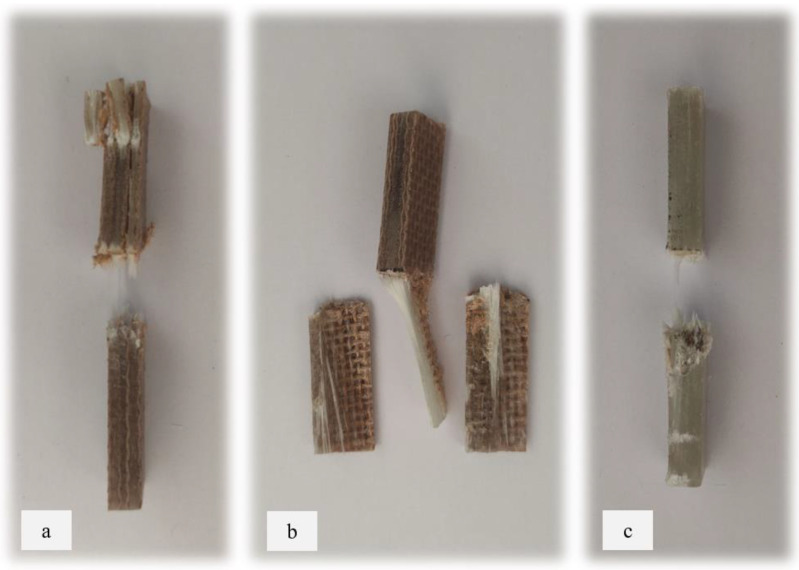
Damage on samples after Izod impact testing, (**a**) pultruded WK/UG alternate; (**b**) pultruded 3WK/UG/3WK alternate; (**c**) pultruded WG/UG alternate.

Figure 10 illustrates the results of Izod impact testing, clearly depicting the failure modes of different specimens. Among the tested samples, the kenaf/glass hybridised specimen, pultruded WK/UG alternate and the pultruded 3WK/UG3WK specimen showed severe delamination upon impact testing. Additionally, more visible fibre pull-outs were observed in the hybrid specimen. Low-velocity impacts, often imperceptible on the surface, represent a particularly crucial form of loading as they induce internal damage within structures [58]. Delaminations, occurring at specific interfaces within laminates, are a notable outcome of these low-velocity impacts, initiated by subsurface cracks. This damage diminishes structural stiffness, leading to the growth of the damage until an eventual fracture occurs. The observed failure modes in the hybrid specimens can be attributed to the damping properties of kenaf fibres [59]. Kenaf fibres have inherent damping characteristics, contributing to a more ductile failure behaviour when compared to glass fibres in the hybridised specimens. The damping property of kenaf fibres allows them to undergo more significant deformation and energy absorption during impact, leading to delamination and fibre pull-outs as the material redistributes stress to absorb the impact energy [60].

On the other hand, the pultruded fibreglass specimen demonstrated a failure mode that lacked signs of delamination. This is in line with the nature of glass fibres, known for their high tensile modulus and brittle properties. Glass fibres have excellent stiffness and strength but are relatively brittle and more prone to sudden fracture under impact loads. As a result, the failure observed in the fibreglass specimen was more brittle, with less delamination than the hybridised specimens. The absence of delamination in the fibreglass specimen can be attributed to the higher stiffness and reduced damping capacity of glass fibres. Unlike kenaf fibres, glass fibres are less deformable and have lower energy absorption capabilities. Consequently, when subjected to impact forces, glass fibres tend to experience sudden fracture and minimal deformation, leading to brittle failure with limited delamination.

Finding the right balance between kenaf and glass fibres in hybrid composites is vital to achieve the desired combination of properties. Considering factors such as specific application requirements and the trade-off between weight reduction and impact performance is essential when selecting the appropriate kenaf-to-glass ratio for a particular composite application.

### 3.4. Density Analysis

One key factor that plays a crucial role in defining the characteristics of a composite material is its density. The density determines whether the material can offer improved strength-to-weight or cost-to-weight ratios for designing various applications. This aspect is critical in pultruded profile applications due to the advantages of lighter structural products. Lighter profiles are easier to handle and transport, making them more convenient for installation, assembly, and maintenance. They are less cumbersome and pose a lower risk of injury during handling and installation. Besides that, in applications such as aerospace, automotive, and renewable energy sectors, lighter pultruded profiles contribute to lower energy consumption, leading to enhanced fuel efficiency, extended battery life, and optimised system performance.

The average density values obtained from density tests from highest to lowest are pultruded fibreglass (1.6697 g/cm^3^), followed by pultruded kenaf/glass alternate (1.5101 g/cm^3^) and pultruded 3WK/UG/3WK (1.3504 g/cm^3^). Pultruded WK/UG alternate recorded a decrease in density values at about 9.56% when compared to pultruded WG/UG alternate. The density is significantly reduced when pultruded 3WK/UG/3WK recorded 19.12% lower compared to the pultruded WG/UG alternate. It is evident that as the ratio of kenaf-to-glass fibre loading increases, the density of the pultruded composites decreases, resulting in lighter materials.

### 3.5. Specific Strength, Cost, and Weight Analysis

**Figure 11:**
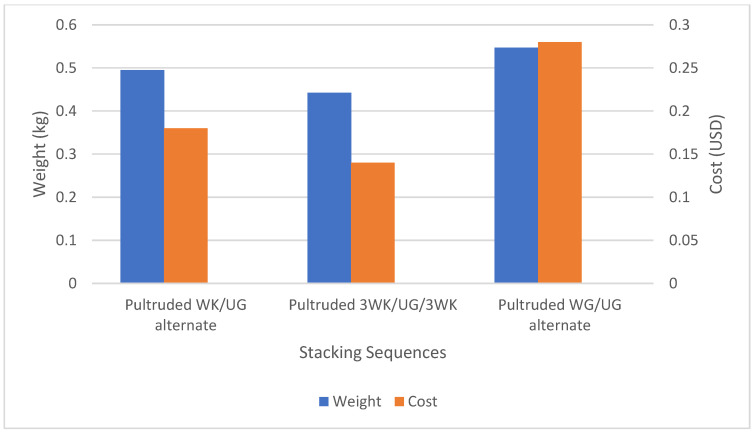
Cost of fibres and weight comparison of 0.3048 m (1 ft) pultruded hollow square tube.

The weight and cost analysis were conducted for 0.3048 m (1 ft) of kenaf/glass reinforced pultruded hollow square tubes (Figure 11). The weight rankings, from heavy to lightweight, were determined as follows: pultruded WG/UG alternate ranked heaviest, followed by pultruded WK/UG alternate, and finally, pultruded 3WK/UG/3WK as the lightest among the three. The pultruded WK/UG alternate recorded a weight that was 9.6% lower than the pultruded WG/UG alternate. This reduction in weight highlights the advantage of incorporating kenaf fibre in the pultruded profile, which contributes to a more lightweight structural component. Furthermore, the pultruded 3WK/UG/3WK profile demonstrated an even more significant weight reduction of 19.1% compared to the conventional fibreglass profile. This substantial decrease in weight signifies the significant benefits of adopting plant fibres like kenaf in pultruded profiles, offering the potential for lightweight structural parts in various applications.

Similarly, the cost analysis resulted in rankings from highest to lowest cost as follows: pultruded WG/UG alternate with the highest cost, followed by pultruded WK/UG alternate, and finally, pultruded 3WK/UG/3WK with the lowest cost among the three. The cost data of reinforcing fibres (per kg) in Ringgit Malaysia and US Dollar is listed in Table 6.

**Figure 12:**
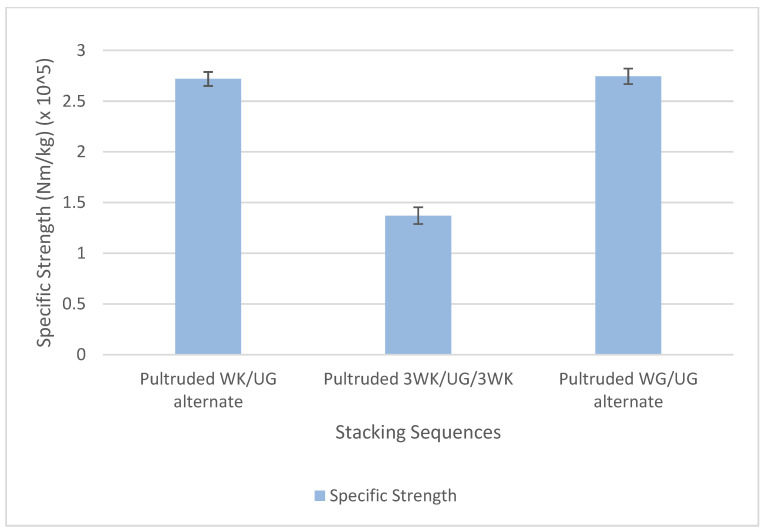
Specific strength comparison.

The pultruded WK/UG alternate exhibited a remarkable cost reduction of 35.7% compared to the pultruded WG/UG alternate. This significant cost reduction showcases the economic advantages of utilising kenaf fibre in pultruded profiles, making it a cost-effective alternative without compromising performance. Moreover, the pultruded 3WK/UG/3WK profile demonstrated an outstanding cost reduction of 50% compared to the conventional pure glass fibre reinforced profile. This remarkable cost-saving potential positions pultruded profiles with plant fibres like kenaf as an attractive option for various industries looking to reduce raw material costs while maintaining structural integrity. The specific strength (strength to weight ratio) of pultruded composites is compared in Figure 12. The pultruded WK/UG alternate recorded a specific strength of 2.719 × 10^5^ Nm/kg, while the pultruded WG/UG alternate recorded 2.745 × 10^5^ Nm/kg, showing only a slight decrease of about 0.95%. Additionally, the specific strength of pultruded 3WK/UG/3WK is about 1.371 × 10^5^ Nm/kg, which is approximately 50.05% lower than that of the pultruded WK/UG alternate.

The specific strength, weight, and cost analysis demonstrates the positive implications of incorporating plant fibres, such as kenaf, into pultruded profiles. Using kenaf contributes to lightweight structural parts, offering potential benefits in weight reduction and enhanced strength performance. Additionally, it significantly lowers the raw material cost, making it a cost-effective and environmentally friendly alternative to traditional fibreglass reinforcement [61,62]. Lighter profiles reduce transportation costs since less energy is needed to move them. This is particularly beneficial when transporting large quantities of profiles or shipping over long distances. They also contribute to environmental sustainability by reducing the consumption of raw materials and energy during manufacturing and transportation. These findings highlight the promising prospects of plant fibre-reinforced pultruded profiles as a viable solution for various industries seeking lightweight and economical structural components.

Based on the conducted study, hybrid pultruded composites have demonstrated their suitability for structural applications, showcasing commendable fatigue and impact properties when compared to traditional pultruded fibreglass. Notably, the hybrid profiles excel in terms of cost and weight, presenting a distinct advantage over conventional pultruded fibreglass composites. The pultruded WK/UG alternate variant exhibited comparable fatigue and impact properties to its fibreglass counterpart. In contrast, the pultruded 3WK/UG/3WK composites achieved substantial weight and cost reduction. Consequently, the WK/UG alternate configuration holds potential for moderate load-bearing structural applications, while the 3WK/UG/3WK variant proves attractive for low load-bearing structural applications due to its significant weight reduction and cost-effectiveness, coupled with satisfactory durability. However, it is imperative to acknowledge the limitations of these hybrid specimens, particularly in their constraint for outdoor applications. Challenges such as moisture sensitivity, susceptibility to biodegradation, limited fire resistance, and vulnerability to UV exposure need to be addressed. To overcome these challenges and further enhance the applicability of plant fibres in outdoor and structural contexts, future research is recommended. This includes the development of advanced coatings and an exploration of hygrothermal properties. These endeavours can contribute to expanding the versatility and resilience of plant fibre composites in diverse environmental conditions.

## 4. Conclusions

Based on the study, the following conclusions can be drawn:

1. The fatigue analysis results reveal that among the investigated pultruded composites, the pultruded WG/UG alternate demonstrates the highest fatigue resistance, followed by the pultruded WK/UG alternate and pultruded 3WK/UG/3WK specimens. However, when the stress level reaches 60% of the ultimate tensile strength (UTS), all the data points are situated within the high-cycle fatigue region. This suggests that the kenaf/glass hybrid pultruded profiles have potential for long-term reliability in low to moderate load structural applications involving cyclic loading.
2. The pultruded WG/UG alternate also exhibits the highest fatigue sensitivity, followed by the pultruded WK/UG alternate and pultruded 3WK/UG/3WK specimens, implying that an increase in the kenaf-to-glass ratio leads to reduced fatigue resistance and sensitivity. The low fatigue sensitivity observed in kenaf/glass hybrid composites signifies the presence of damping properties within these materials, which contributes to the overall reliability, safety, and cost-effectiveness of structural profiles.
3. Interestingly, Izod impact analysis illustrates that the impact strength of pultruded WK/UG alternate hybrid composites aligns closely with that of pultruded WG/UG alternate composites. However, a further elevation in the kenaf to glass fibre ratio in pultruded 3WK/UG/3WK alternate hybrids results in a decline in impact strength. The findings suggest that the pultruded WK/UG alternate profile is able to withstand impact forces without undergoing brittle fractures, which ensures safety and structural integrity.
4. Hybrid specimens, such as pultruded 3WK/UG/3WK, marks a significant reduction in weight and cost followed by the pultruded WK/UG alternate when compared to the pultruded WG/UG alternate. Moreover, an almost similar specific strength has been recorded by the pultruded WK/UG alternate when compared to the pultruded WG/UG alternate. The comprehensive assessment of specific strength, weight, and cost underscores the advantageous potential of incorporating plant fibres in pultruded profiles, contributing to the creation of lightweight structural components while simultaneously reducing raw material costs.
5. Among the hybrid samples investigated, the pultruded WK/UG alternate demonstrated good fatigue and impact properties, proving possibilities in moderate load structural applications. Meanwhile, the pultruded 3WK/UG/3WK exhibited noteworthy advantages in weight and cost reduction, rendering it a suitable choice for low-load structural applications. These findings collectively emphasise the intricate interplay between plant-glass fibre composition, hybridisation, and resultant mechanical properties. Such insights are invaluable for the strategic design and optimisation of plant fibres in pultruded composites for diverse structural applications offering sustainable and cost-effective advantages.
6. While hybrid specimens exhibit notable strengths, their limitations are evident, particularly in outdoor applications. Plant fibres, constituting a significant component of the hybrid, are inherently sensitive to moisture, biodegradation, and UV exposure, and they pose challenges in terms of fire resistance. To overcome these constraints and broaden the spectrum of potential applications, future research should prioritize the development of advanced coatings and hygrothermal research to elevate applications of plant fibres in outdoor structural applications.

## Figures and Tables

**Table 1:** Industries and applications of pultruded profiles [5,6,7].

Industries	Applications
Aerospace and Defence	Aircraft components, such as structural beams, floors, and panels. Interior components, such as overhead bins, seating structures, and cabin partitions. Military vehicle components, such as chassis elements, weapon mounts, and armour supports.
Automotive	Chassis components, such as frame rails, cross members, and subframes. Interior panels, such as door panels, dashboard supports, and seat structures.
Construction	Bridge components, such as beams, decks, and railings. Building facades, such as cladding, sunshades, and louvres. Railing systems, such as stairs, balconies, and elevated platforms.
Marine	Boat and shipbuilding components, such as hull reinforcements, deck supports, bulkheads, and superstructure elements. Decking and Flooring, such as non-slip decking and flooring solutions for marine vessels, docks, and decks.

**Table 2:** Mechanical and physical properties of raw materials.

Properties	Kenaf	E-Glass	Unsaturated Polyester Resin (UPR)
Tensile strength (MPa)	400–930	2000–3500	40–90
Tensile modulus (GPa)	53	70	3.3
Elongation (%)	2.7	2.8	2
Density (g/cm^3^)	1.2	2.5	1.2
Linear density (tex)	400–500	2400	-
Filament diameter (µm)	60–80	24	-
Weight of woven mat (g/m^2^)	375	450	-

**Table 3:** Stacking sequence of pultruded kenaf/glass hybrid composites and pure fibreglass composites.

Samples	Stacking Sequence	Fibre Weight Percentage (wt.%)	
		Kenaf Content	Glass Content
Pultruded WK/UG alternate	WK/UG/WK/UG/WK/UG/WK	45	55
Pultruded 3WK/UG/3WK	WK/WK/WK/UG/WK/WK/WK	81.7	18.3
Pultruded WG/UG alternate	WG/UG/WG/UG/WG/UG/WG	0	100

WK—woven kenaf. WG—woven glass. UG—unidirectional glass roving.

**Table 4:** Applied Stress on Fatigue Testing.

Type of Composites	Ultimate Tensile Strength (UTS), MPa	Coefficient of Variation, %	Percentage of Applied Load with Respect to UTS, %	Maximum Stress (Q_max_), MPa	Minimum Stress (Q_min_), MPa
Pultruded WK/UG alternate	410.6	2.54	80	328.48	32.85
			70	287.42	28.74
			60	246.36	24.64
			50	205.3	20.53
Pultruded 3WK/UG/3WK	185.13	5.17	80	148.10	14.81
			70	129.59	12.96
			60	111.08	11.11
			50	92.57	9.26
Pultruded WG/UG alternate	458.28	2.77	80	366.62	36.66
			70	320.8	32.08
			60	274.97	27.5
			50	229.14	22.91

**Table 5:** Damage on samples after fatigue testing.

Stress Intensity	50%	60%	70%	80%
Pultruded WK/UG alternate	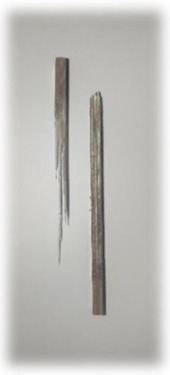	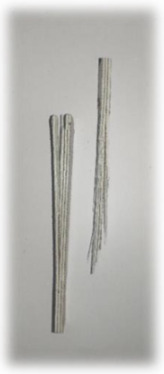	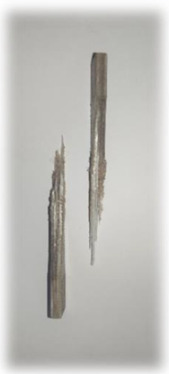	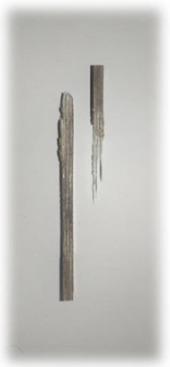
Pultruded 3WK/UG/3WK	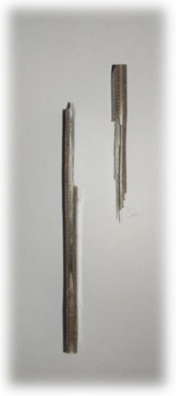	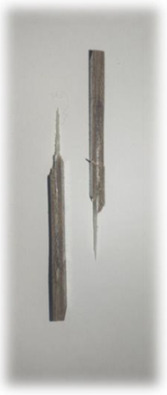	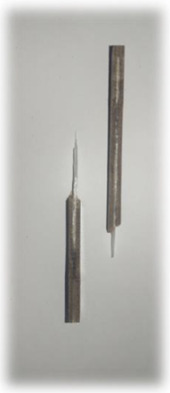	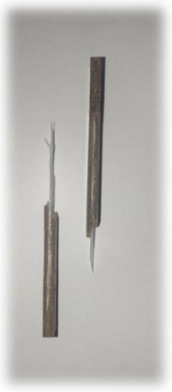
Pultruded WG/UG alternate	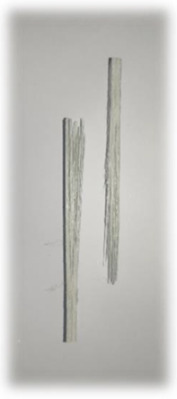	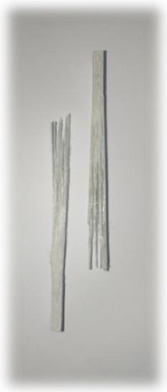	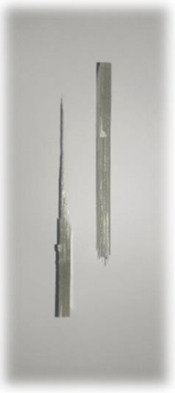	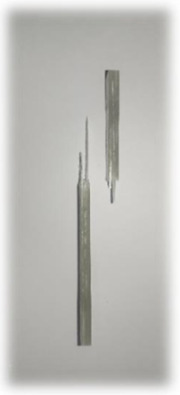

**Table 6:** Cost of Reinforcing Fibres in Malaysia.

Reinforcing Fibres	Cost per kg in Ringgit Malaysia (RM)	Cost Per kg in US Dollar (USD)
Glass fibre direct roving	3.50	0.75
Woven glass fibre mat	5.20	1.12
Woven kenaf fibre mat	2.50	0.54

The cost data presented reflects values in USD converted from Malaysian Ringgit and is based on rates in Malaysia as of November 2023.

## Data Availability

The authors confirm that the data supporting the findings of this study are available within the article.

## References

[1] Minchenkov K., Gusev S., Sulimov A., Sergeichev I., Safonov A. (2023). Experimental and Numerical Analyses of the Thermoplastic Pultrusion of Large Structural Profiles. Mater. Des..

[2] Volk M., Yuksel O., Baran I., Hattel J.H., Spangenberg J., Sandberg M. (2022). Cost-Efficient, Automated, and Sustainable Composite Profile Manufacture: A Review of the State of the Art, Innovations, and Future of Pultrusion Technologies. Compos. B Eng..

[3] Esfandiari P., Silva J.F., Novo P.J., Nunes J.P., Marques A.T. (2022). Production and Processing of Pre-Impregnated Thermoplastic Tapes by Pultrusion and Compression Moulding. J. Compos. Mater..

[4] Sandberg M., Yuksel O., Baran I., Hattel J.H., Spangenberg J. (2021). Numerical and Experimental Analysis of Resin-Flow, Heat-Transfer, and Cure in a Resin-Injection Pultrusion Process. Compos. Part A Appl. Sci. Manuf..

[5] Correia J.R. (2023). Pultrusion of Advanced Composites. Advanced Fibre-Reinforced Polymer (FRP) Composites for Structural Applications.

[6] Vedernikov A., Safonov A., Tucci F., Carlone P., Akhatov I. (2020). Pultruded Materials and Structures: A Review. J. Compos. Mater..

[7] Barkanov E., Akishin P., Namsone E., Auzins J., Morozovs A. (2021). Optimization of Pultrusion Processes for an Industrial Application. Mech. Compos. Mater..

[8] Chethan N., Nagesh S.N., Sunith Babu L. (2021). Mechanical Behaviour of Kenaf-Jute-E-Glass Reinforced Hybrid Polymer Composites. Mater Today Proc..

[9] Baeza C., Jesús P., Franco H., Cortés P., Bele E., Agaliotis E.M., Gemi L., Madenci E., Özkılıç Y.O., Yazman S. (2022). Effect of Fiber Wrapping on Bending Behavior of Reinforced Concrete Filled Pultruded GFRP Composite Hybrid Beams. Polymers.

[10] Vayabari D.A.G., Ilham Z., Md Saad N., Usuldin S.R.A., Norhisham D.A., Abd Rahim M.H., Wan-Mohtar W.A.A.Q.I. (2023). Cultivation Strategies of Kenaf (*Hibiscus cannabinus* L.) as a Future Approach in Malaysian Agriculture Industry. Horticulturae.

[11] Al-Mamun M., Rafii M.Y., Misran A.B., Berahim Z., Ahmad Z., Khan M.M.H., Oladosu Y. (2023). Heterosis and Combining Ability Estimate on Yield and Yield-Related Traits in a Half Diallel Crosses of Kenaf (*Hibiscus cannabinus* L.) in Malaysia. J. Nat. Fibers.

[12] Deepa C., Rajeshkumar L., Ramesh M. (2021). Thermal Properties of Kenaf Fiber-Based Hybrid Composites. Natural Fiber-Reinforced Composites: Thermal Properties and Applications.

[13] Prem Kumar R., Muthukrishnan M., Felix Sahayaraj A. (2023). Effect of Hybridization on Natural Fiber Reinforced Polymer Composite Materials—A Review. Polym. Compos..

[14] Muralidharan N.D., Subramanian J., Rajamanickam S.K., Gopalan V. (2023). An Experimental Investigation of Flame Retardancy and Thermal Stability of Treated and Untreated Kenaf Fiber Reinforced Epoxy Composites. J. Polym. Eng..

[15] Zakaria N.A., Ishak M.R., Mustapha F., Yidris N. (2023). Tensile Properties of a Hybrid Kenaf-Glass Fibre Composite Shaft. Mater. Today Proc..

[16] Fernandes O., Dutta J., Pai Y. (2023). Effect of Various Factors and Hygrothermal Ageing Environment on the Low Velocity Impact Response of Fibre Reinforced Polymer Composites—A Comprehensive Review. Cogent Eng..

[17] Grzejda R., Warzecha M., Urbanowicz K. (2022). Determination of the Preload of Bolts for Structural Health Monitoring of a Multi-Bolted Joint: FEM Approach. Lubricants.

[18] Bhowmik S., Kumar S., Mahakur V.K. (2023). Various Factors Affecting the Fatigue Performance of Natural Fiber-Reinforced Polymer Composites: A Systematic Review. Iran. Polym. J..

[19] Wang Z., Xian G. (2023). Impact Performances of Fiber Reinforced Polymer Composites and Cables: A Review. Compos. Struct..

[20] Asumani O., Paskaramoorthy R. (2020). Fatigue and Impact Strengths of Kenaf Fibre Reinforced Polypropylene Composites: Effects of Fibre Treatments. Adv. Compos. Mater..

[21] Feng N.L., DharMalingam S., Zakaria K.A., Selamat M.Z. (2017). Investigation on the Fatigue Life Characteristic of Kenaf/Glass Woven-Ply Reinforced Metal Sandwich Materials. J. Sandw. Struct. Mater..

[22] Miah M.S., Yu J., Yang Y., Memon H., Rashid M.A. (2021). Durability and Notch Sensitivity Analysis of Environmental Ageing Induced Glass Fibre Mat and Kenaf Fibre Mat-Reinforced Composites. J. Ind. Text..

[23] Supian A.B.M., Sapuan S.M., Jawaid M., Zuhri M.Y.M., Ilyas R.A., Syamsir A. (2022). Crashworthiness Response of Filament Wound Kenaf/Glass Fibre-Reinforced Epoxy Composite Tubes with Influence of Stacking Sequence under Intermediate-Velocity Impact Load. Fibers Polym..

[24] Owen M.M., Achukwu E.O., Arukalam I.O., Romli A.Z. (2022). Effect of Varying Processing Temperatures on the Mechanical and Microstructural Properties of Kenaf Fibre-ABS Composites for Moderate Temperature Applications. Polym. Renew. Resour..

[25] Al-Waily M., Mechi S.A. (2021). Fatigue Characterizations Modifying for Below Knee Prosthesis Composite Materials by Using Natural Knitted Kenaf Reinforcement Fibers. Int. J. Energy Environ..

[26] Xian G., Guo R., Li C. (2022). Combined Effects of Sustained Bending Loading, Water Immersion and Fiber Hybrid Mode on the Mechanical Properties of Carbon/Glass Fiber Reinforced Polymer Composite. Compos. Struct..

[27] Guo R., Xian G., Li C., Hong B. (2022). Effect of Fiber Hybrid Mode on the Tension–Tension Fatigue Performance for the Pultruded Carbon/Glass Fiber Reinforced Polymer Composite Rod. Eng. Fract. Mech..

[28] Tang J., Zhou Z., Chen H., Wang S., Gutiérrez A. (2022). Research on the Lightweight Design of GFRP Fabric Pultrusion Panels for Railway Vehicle. Compos. Struct..

[29] Duchet-Rumeau J., Gérard J.-F., Barkanov E., Akishin P., Namsone-Sile E. (2022). Effectiveness and Productivity Improvement of Conventional Pultrusion Processes. Polymers.

[30] Alsinani N., Laberge Lebel L. (2022). Effect of High Pulling Speeds on the Morphologies of Pultrudates in a Thermoplastic Pultrusion Process. J. Thermoplast. Compos. Mater..

[31] Strauss S., Wilhelm F., Senz A., Engelen H., Boysen S., Rilli N., Celik A., Ratka M., Bonten C. (2023). Experimental and Simulative Analysis of the Pressure Development in a Closed Injection Pultrusion Process with Multiple Chamber Geometries. Polymers.

[32] Setyanto D., Antonio Y.A., Darmawan M., Ubaidillah U. (2022). A Novel Z Profile of Pultruded Glass-Fibre-Reinforced Polymer Beams for Purlins. Sustainability.

[33] Cheng K., Wang Y., Fang H., Qian C., Wu P. (2023). Experimental Investigation and Prediction for Bending Creep of Glass Fiber-Reinforced Polymer Pultruded Tube. Buildings.

[34] Khalilabad H., Ruiz Emparanza E., De Caso A., Roghani F., Khodadadi H., Nanni N., Khalilabad E.H., Ruiz Emparanza A., De Caso F., Roghani H. (2023). Characterization Specifications for FRP Pultruded Materials: From Constituents to Pultruded Profiles. Fibers.

[35] Badaruzzaman W.H.W., Dabbagh N.M.R., Salleh K.M., Saharuddin E.N., Radzi N.F.M., Azham M.A.A., Sani S.F.A., Zakaria S. (2022). Mechanical Properties and Water Absorption Capacity of Hybrid GFRP Composites. Polymers.

[36] Ramraji K., Rajkumar K., Harikrishna K.L., Sarmaji Kumar P. (2022). Mechanical and Dynamic Mechanical Analysis of Calcium Carbonate Filler Interleaved with Basalt Polymeric Laminates. Mater. Today Proc..

[37] Hazwani M., Abdul Majid M.S., Azaman M.D., Ridzuan M.J.M., Cheng E.M. (2023). Mechanical Properties and Flammability of Pineapple Leaf Fiber (PALF) Reinforced Polymer Composite with Hybridized Fire Retardants. Mater. Today Proc..

[38] Gupta A., Vaishya R., Khan K.L.A., Walia R.S., Singh H. (2019). Multi-Response Optimization of Hybrid Filler Composition for Pultruded Jute Fiber Reinforced Polymer Composite. Mater. Res. Express.

[39] Vedernikov A., Nasonov Y., Korotkov R., Gusev S., Akhatov I., Safonov A. (2021). Effects of Additives on the Cure Kinetics of Vinyl Ester Pultrusion Resins. J. Compos. Mater..

[40] Sharba M.J., Leman Z., Sultan M.T.H., Ishak M.R., Hanim M.A.A. (2016). Partial Replacement of Glass Fiber by Woven Kenaf in Hybrid Composites and Its Effect on Monotonic and Fatigue Properties. Bioresources.

[41] Liang S., Gning P.B., Guillaumat L. (2012). A Comparative Study of Fatigue Behaviour of Flax/Epoxy and Glass/Epoxy Composites. Compos. Sci. Technol..

[42] Hassan F., Zulkifli R., Ghazali M.J., Azhari C.H. (2017). Kenaf Fiber Composite in Automotive Industry: An Overview. Int. J. Adv. Sci. Eng. Inf. Technol..

[43] Asyraf M.R.M., Rafidah M., Azrina A., Razman M.R. (2021). Dynamic Mechanical Behaviour of Kenaf Cellulosic Fibre Biocomposites: A Comprehensive Review on Chemical Treatments. Cellulose.

[44] Sivakumar D., Ng L.F., Lau S.M., Lim K.T. (2018). Fatigue Life Behaviour of Glass/Kenaf Woven-Ply Polymer Hybrid Biocomposites. J. Polym. Environ..

[45] Hadiji H., Assarar M., Zouari W., Pierre F., Behlouli K., Zouari B., Ayad R. (2020). Damping Analysis of Nonwoven Natural Fibre-Reinforced Polypropylene Composites Used in Automotive Interior Parts. Polym. Test..

[46] Diharjo K., Susilo D.D., Sudargo P.H., Kaleg S. (2018). Vibration-Damping Factor of Glass/Kenaf/Polyester Hybrid Composite. Key Eng. Mater..

[47] Ramraji K., Rajkumar K., Subbiah M., Balachandar K., Sarmaji Kumar P. (2022). Stacking Layer Effect on Mechanical and Vibration Behaviour of Woven Glass Intertwined with Kenaf Fiber Polymeric Composites. Mater. Today Proc..

[48] Sharba M.J., Leman Z., Sultan M.T.H., Ishak M.R., Hanim M.A.A. (2015). Monotonic and Fatigue Properties of Kenaf /Glass Hybrid Composites under Fully Reversed Cyclic Loading. IOP Conf. Ser. Mater. Sci. Eng..

[49] Sharba M.J., Leman Z., Sultan M.T.H., Ishak M.R., Azmah Hanim M.A. (2016). Effects of Kenaf Fiber Orientation on Mechanical Properties and Fatigue Life of Glass/Kenaf Hybrid Composites. Bioresources.

[50] Arora S., Chitkara R., Dhangar A.S., Dubey D., Kumar R., Gupta A. (2022). A Review of Fatigue Behavior of FRP Composites. Mater. Today Proc..

[51] Noël M. (2019). Probabilistic Fatigue Life Modelling of FRP Composites for Construction. Constr. Build. Mater..

[52] Gao Q., Xin H., Correia J.A.F.O., Mosallam A.S., Berto F. (2022). Probabilistic Fatigue Life Analysis Considering Mean Stress Effects of Fiber Reinforced Polymer (FRP) Composites. Int. J. Fatigue.

[53] Kim J.H., Kwon D.J., Lim C.S., Seo B.K., DeVries K.L., Park J.M. (2023). Interfacial Adhesion Evaluation via Wettability for Fiber Reinforced Polymer Composites: A Review. Compos. Interfaces.

[54] Khan A., Sapuan S.M., Siddiqui V.U., Zainudin E.S., Zuhri M.Y.M., Harussani M.M. (2023). A Review of Recent Developments in Kenaf Fiber/Polylactic Acid Composites Research. Int. J. Biol. Macromol..

[55] Singh K., Das D., Nayak R.K., Khandai S., Kumar R., Routara B.C. (2020). Effect of Silanizion on Mechanical and Tribological Properties of Kenaf-Carbon and Kenaf-Glass Hybrid Polymer Composites. Mater. Today Proc..

[56] Saroj S., Nayak R.K. (2021). Improvement of Mechanical and Wear Resistance of Natural Fiber Reinforced Polymer Composites Through Synthetic Fiber (Glass/Carbon) Hybridization. Trans. Indian Inst. Met..

[57] Ghani M.U., Siddique A., Abraha K.G., Yao L., Li W., Khan M.Q., Kim I.S. (2022). Performance Evaluation of Jute/Glass-Fiber-Reinforced Polybutylene Succinate (PBS) Hybrid Composites with Different Layering Configurations. Materials.

[58] Sadighi M., Alderliesten R. (2022). Impact Fatigue, Multiple and Repeated Low-Velocity Impacts on FRP Composites: A Review. Compos. Struct..

[59] Yusoff R.B., Takagi H., Nakagaito A.N. (2023). A Comparative Study of Polylactic Acid (PLA)-Based Unidirectional Green Hybrid Composites Reinforced with Natural Fibers Such as Kenaf, Bamboo and Coir. Hybrid Adv..

[60] Zhang J., Khatibi A.A., Castanet E., Baum T., Komeily-Nia Z., Vroman P., Wang X. (2019). Effect of Natural Fibre Reinforcement on the Sound and Vibration Damping Properties of Bio-Composites Compression Moulded by Nonwoven Mats. Compos. Commun..

[61] Sathish S., Karthi N., Prabhu L., Gokulkumar S., Balaji D., Vigneshkumar N., Ajeem Farhan T.S., Akilkumar A., Dinesh V.P. (2021). A Review of Natural Fiber Composites: Extraction Methods, Chemical Treatments and Applications. Mater. Today Proc..

[62] Rasheed R., Anwar I., Tahir F., Rizwan A., Javed H., Sharif F. (2023). Techno-Economic and Environmental Sustainability Analysis of Filament-Winding versus Pultrusion Based Glass-Fiber Composite Technologies. Environ. Sci. Pollut. Res..

